# Probing the binding hypothesis of Smad3 modulators by molecular dynamic simulations for Atherosclerosis Cardiovascular Disease (ASCVD)

**DOI:** 10.1371/journal.pone.0324677

**Published:** 2025-06-04

**Authors:** Ubair Aziz, Ishrat Jabeen

**Affiliations:** Computational Drug Design Lab, School of Interdisciplinary Engineering & Sciences (SINES), National University of Science & Technology (NUST), Islamabad, Pakistan; University of Louisville, UNITED STATES OF AMERICA

## Abstract

Transforming Growth Factor β (TGFβ) pathway has been recognized as one of the major processes involved in fibrotic diseases including the Atherosclerosis Cardiovascular Disease (ASCVD). Many drugs have been proposed and are under clinical trials for modulation of the TGFβ pathway by targeting TGFβ receptor. Recently, various proof of the concept studies have advocated that the inhibition of TGFβ-mediated Smad pathway could produce more focused effect with less off target toxicities in ASCVD. As these studies lack the mechanism and binding profile of Smad3 modulators, characterization of binding pattern for Smad3 inhibitors can provide a platform for the lead optimization against ASCVD. We utilized dimeric inhibitors from the PubChem dataset (PubChem ID: 630) of Smad3-FoxH1 binding inhibitors to generate binding hypothesis of Smad3 inhibitors. The selected compounds from the dataset were docked and ligand-protein complexes were simulated for 250 ns for further sampling of conformational space and to obtain stable binding hypothesis. Root Mean Square Deviation (RMSD), Root Mean Square Fluctuation (RMSF) and hydrogen bond analysis of ligand-protein complexes after simulations revealed that Asn320 in Smad3 provides an efficient inhibition site for the two most potent small inhibitors (hereby named **SM1** and **SM2**) of Smad3-FoxH1 binding. Although the diverse nature of compounds produce variable interaction patterns with FoxH1 binding site in Smad3, Gln315, Gln364 and Arg367 were observed to be the most common hydrogen bond interaction points in this binding site. Additionally, two compounds (hereby named **SM8** and **SM19)** detached from the FoxH1 binding site and formed a highly stable complex around Tyr323 via hydrophobic complementarity, suggesting a new binding site for modulation of Smad3 activity.

## Introduction

Atherosclerotic Cardiovascular Diseases (ASCVD) are the most lethal diseases across the globe. These accounted for more than 18 million fatalities in 2019 holding the top three positions for most deaths in 2019 [1]. Vascular fibrosis is accepted as one of the leading cause of atherosclerosis that can be aggravated by other fibrogenic processes such as hypertension, hyperglycemia, dyslipidemia and hyperhomocysteinemia [2]. Pathogenic factors for vascular fibrosis include Rennin-Angiotensin-Aldosterone System (RAAS), Transforming Growth Factor β (TGFβ) signaling, Connective Tissue Growth Factor (CTGF) proteins, Matrix Metallo-Proteins (MMPs) and Peroxisome Proliferator Activated Receptors (PPARs) [2].

Among these factors, RAAS has been extensively targeted as hypertension management medication with Angiotensin Converting Enzyme (ACE) inhibitors and Angiotensin Receptor Blockers (ARBs). However, these ACE inhibitors and ARBs pose the risk of angioedema [3,4]. Similarly, statins (the inhibitors of HMG-CoA) also show quite promising results by lowering the LDL-C (Low Dietary Lipoprotein C) levels in blood consequently preventing thrombus formation. However, apart from the anti-inflammatory actions of statins [5], these have been shown to mediate adipose tissue inflammation and insulin resistance. Like, prompting an increase in risk of diabetes [6].

TGFβ signaling regulates a range of essential biological processes including body axis formation, embryonic development, immune regulation, tissue homeostasis and wound healing. These responses are carried out either via Smad-dependent ubiquitous pathway (termed as canonical pathway) or through some tissue specific intracellular (non-canonical) signaling cascade like small GTPases (Ras, Raf), Mitogen Activated Protein Kinases (MAPK), and Phasphatidylinositol 3 Kinases (PI3K) [7]. Previously, TGFβ signaling has been extensively studied for its role in cardiovascular diseases and cancer [8–11]. Consequently, various TGFβ receptor inhibitors such as vactosertib [12] and galunisertib [13] are in clinical trials [14] to manage TGFβ-related diseases. Almost all of these drugs target TGFβ receptor and inhibit either binding of TGFβ to receptor or the receptor activity [14]. Although the significance of Smad3 protein of TGFβ pathway in ASCVD has been demonstrated in various genetic and knock down studies [15–24], development of non-toxic Smad3 inhibitors have been a challenge due to unknown mechanism and toxicity of inhibitors. SIS3, a potent inhibitor of Smad3 phosphorylation, has been used in research for over a decade [25–27] but fails to qualify as a drug owing to its low water and high lipid solubility (cLogp = 4.7) that attributes to its poor pharmacokinetic properties and difficulties in drug formulation [28]. Additionally, no information regarding mechanism of action or binding pattern of SIS3 is available that can contribute toward development of drugs with better toxicity profiles. In this regard, a binding hypothesis against Smad3 for its inhibition with pharmacokinetic properties can act as a scaffold for lead optimization.

To fill this research gap, we aim to establish a consensus binding pattern of Smad3 inhibitors, providing a valuable foundation for the development of Smad3-modulating drugs for conditions such as ASCVD. The stable binding interactions identified in this research could serve as a crucial platform for designing potential drug candidates targeting ASCVD.

## Materials and methods

### Protein model preparation and molecular dynamic simulation

The three dimensional (3D) crystallographic structures of Smad3 protein in human were obtained from Protein Data Bank (PDB). As MH2 domain mediates the activity of Smad3, 3D structure of only MH2 domain were considered for this study. Among the available 3D crystal structures (S1 Table), only two structures contained Smad3 MH2 domain in free state (PDB IDs: 1mjs and 1mk2). 1mjs structure was of higher resolution (1.91Å) but contained missing regions from residue 323–327 and 380–386 while 1mk2 has a complete structure of MH2 domain at a comparatively lower resolution (2.74Å). To obtain a high-quality 3D structure, homology models of MH2 domain was constructed with 1mjs as initial template and 1mk2 to model only the missing regions.

Ten homology models of Smad3 MH2 domain were generated using Modeller 10.1 [29] and the one having best DOPE (Discrete Optimized Protein Energy) score [30] was selected. DOPE is a knowledge-based potential that is trained on the known protein structures available in PDB to measure the native-likeliness of folds in modelled structure using the equation (i).


$$Dope\;Score=\sum_{i<j}w_{i,j}u(d_{ij})$$ (i)

where

$w_{i,j}$ = weight factor accounting for environment as well as sequence separation of atoms *i* and *j.*

$\left(d_{ij}\right)$ = statistical potential depending upon the atomic distances of atom *i* and *j*. The function *u(d*_*ij*_*)* is a derivation of probability equation for finding the atomic pair (*i* and *j*) at a specific distance using the equation (ii).


$$u(d)={-k}_{B}T\ln\frac{P_{obs}(d)}{P_{ref}(d)}$$ (ii)

where

*k*_*B*_ = Boltzmann constant

*T* = Temperature

*P*_*obs*_*(d)* = Observed probability distribution of interatomic distances in known proteins

*P*_*ref*_*(d)* = Reference probability distribution, representing the idealized state

Additional evaluation of structure was done by Prosa-Web server [31] and Ramachandran plot [32]. In order to evaluate the structural stability, the modelled structure was then simulated for 50 ns in a water filled (TIP3P) cubical box with margins of the box having a distance of at least 1.0nm from protein using AMBER99SB ILDN force field [33] in GROMACS 2019.6 [34] to obtain a stable conformation of protein in aqueous solution. Root Mean Square Deviation (RMSD) of ligand heavy atoms from their initial coordinates with reference to protein back bone and Root Mean Square Fluctuation (RMSF) of protein residues were calculated to evaluate the stability of protein and residues within protein respectively during the simulation period. The c-terminal region of Smad3 has an important function in activation of Smad3 via phosphorylation by interacting with TGFβR for initiation of cellular response [35]. However, our study is aimed toward binding inhibition of structurally active Smad3 MH2 domain with transcription factors such as FoxH1. Considering the facts that c-terminal region is not participating in interaction with FoxH1 [36], and the residues in this region showed high RMSF, the c-terminal residues from Gly414 to Ser424 were removed before docking analysis. This truncated protein model was then used to dock compounds prepared in ligand preparation step.

### Ligand data collection and preparation

Till date 16 different studies have reported TGFβ/Smad3 inhibitors (S2 Table). Out of these datasets, only two studies reported greater than 10,000 compounds tested against TGFβ/Smad3. The largest dataset (Pubchem ID: 588855) contained 407,798 compounds but these inhibitors might not have been specifically binding with Smad3 for inhibition as the study was aimed for detection of suppression of Smad3 binding with DNA. This could be accomplished by inhibition of a range of proteins involved in TGFβ/Smad3 pathway. Therefore, the second dataset containing 88,061 compounds that were tested specifically for their inhibitory potential against Smad3-FoxH1 binding at a fixed 25µM concentration was selected (Pubchem ID: 630) [37] for this study. This data can be utilized to explore the interaction patterns and spatial features of Smad3 inhibitors. Out of other 14 remaining datasets, seven studies aimed on binding of Smad3 with Smad Binding Element (SBE) on DNA as an indicator of Smad3 activity with less than 10,000 compounds tested in each study. Remaining six studies focused on inhibitors of Smad3 phosphorylation tested via western blot assay after the treatment of up to three compounds in each study. Only one study aimed towards the inhibition of Smad3 FoxH1 binding inhibition of 37 compounds with only one active inhibitor reported. Herein, we aim to generate the binding hypothesis of Smad3 FoxH1 inhibition interface through a consensus binding patterns among structurally diverse compounds tested through same biological protocol. For this purpose, we selected the data from Smad3-FoxH1 inhibition study (Pubchem ID: 630) [37] containing larger number of Smad3 inhibitors with potentially similar binding pattern. Selected dataset comprised of only 251 active inhibitors of Smad3-FoxH1 binding, out of all 88061 tested compounds, based upon their ability to reduce the binding affinity of Smad3 and FoxH1 by at least 50% as per equation iv. This data was further refined by removing the duplicates and missing values. The Racemic compounds might provide misleading activity values as either one of the enantiomers could have high activity while the other one could have minimal contribution to the activity [38]. Thus, such compounds were removed from the dataset. Furthermore, compounds having molecular weight <200 were also removed to avoid fragments [39]. As the drugs approved during 2010–2020 have a mean molecular weight of 452 with standard deviation of 116 [39]. Remaining compounds were cleaned from any salts or ions present with them to isolate the main component and then the protonation states of charged atoms were neutralized using PyMOL [40]. It was observed that the remaining data of 188 compounds included entries with either complete or partial two-fold symmetry suggesting a symmetrical binding site in protein. Thus, 33 compounds with either perfect or partial two-fold symmetry were selected for this study (S3 Table). Selected compounds were energy minimized to generate 3D stable conformations. These conformations were then used for molecular docking. The overview of data collection and preparation regarding ligands can be visualized in (S1 Fig).

The structures were labeled from **SM1** to **SM33** in the descending order of their activity. Among these compounds, it was observed that all compounds contain at least one homocyclic or heterocyclic hexene ring at both sides of symmetry. In majority of highly active compounds (activity >79.24%), the dimeric substitution units were connected by a linker of two bond distance (**SM1**, **SM2**, **SM3**, **SM5**, **SM9**, **SM15**, **SM17** and **SM20**). These ligands were categorized as ‘Group-I ligands’ (S4 Table). Most of these compounds were observed to carry a hydrogen bond donor (-NH_2_ or -OH) group on each of side of the dimer (**SM1**, **SM2**, **SM5** and **SM9**). In some other compounds, dimeric units were connected by a linker of one bond distance (**SM4**, **SM11**, **SM18**, **SM22**, **SM23** and **SM24**). These ligands were categorized as ‘Group-II ligands’. Among these compounds, again **SM4**, **SM18** and **SM24** have -OH groups on each ringside of the dimer. These structural similarities among active inhibitors suggest that the placement of hydrogen bond donors at the specific distance could be one of the features that mediates their binding with Smad3. Other compounds that have these dimeric scaffolds at more than two bond distance were categorized as ‘Group-III ligands’.


$$FRET\;signal=\frac{F665}{F620}\times 1000$$ (iii)

$$\;\%\;inhibition\;=100-\left(\frac{FRETcompound-FRETpeptide}{FRETcontrol-FRETpeptide}\right)\times 100$$ (iv)

● *FRETcontrol* is FRET signal with 6x-His-Smad3 with Dy-FoxH1-peptide
● *FRETcompound* is FRET signal with compounds at 25µM with Dy-FoxH1-peptide
● *FRETpeptide* is FRET signal with only Dy-FoxH1-peptide

### Molecular docking analysis

FoxH1 binds at the hydrophobic surface of H2, H3 and H4 helices in MH2 domain of Smad3 and specifically interacts with Cys331, Cys337, Leu339, Gln357, Gln364, Arg367, Ala381 and Glu382 via hydrogen bonds [36]. Four cavities were detected in Smad3 protein with a volume >10Å by sphere based cavity detection method with sphere radius of 1.4Å and grid resolution of 0.8Å in Molegro Virtual Docker6.0 [41].The largest cavity had a volume of 25.6Å and surface area of 101.12Å. This cavity was present in region lying between H2 and H4 helix that overlaps with the Smad3 residues that interact with FoxH1 [36]. This binding cavity was selected as site for docking of selected compounds with a 15Å radius of binding site from the center of cavity (Fig a in S2 Fig). 50 runs of docking simulations were performed for each compound using grid resolution of 0.3Å and MolDock scoring function [42] as described in equations below.


$$E_{score}=E_{inter}+E_{intra}$$ (v)

$$E_{inter}=\;\sum_{i\in \;ligand}\sum_{j\in \;protein}\left[E_{PLP(r_{ij})}+332.0\frac{q_{i}q_{j}}{{4r}_{{ij}^{2}}}\right]$$ (vi)

$$E_{intra}=\;\sum_{i\in \;ligand}\sum_{j\in \;ligand}E_{PLP(r_{ij})\;}+\;\sum_{flexible\;bonds}A\left[1-\cos\left(m.\theta -\;\theta_{0}\right)\right]+\;E_{clash}$$ (vii)

MolDock Scores is Pairwise Linear Potential (PLP) derived scoring function that uses two energy terms, E_inter_ and E_intra_ (equation v). E_inter_ calculates the ligand protein interaction energy including electrostatic interactions and E_plp_ that describes the van dar Waals interactions and hydrogen Bond potential (equation vi). E_intra_ calculates the E_plp_ between pair of bound atoms, torsional energy and E_clash_ that assigns a penalty of 1000 if two atoms (more than two bonds apart) have a distance <2.0Å (equation vii) [42].

### Molecular dynamic simulations

The top scoring poses from each docked compound were used for hydrogen bond stability and RMSD analysis using Molecular Dynamic (MD) simulation. For MD simulations, AMBER99SB ILDN [33] force field was used in GROMACs 2019.6 [43]. The atomic parameters for ligands were generated by ACPYPE (AnteChamber Python Parser Interface) 2022.3.11 [44–46]. A triclinic box was generated around protein with a minimum of 1.0nm distance between protein atoms and margins of grid box. Box was filled with three point water models (TIP3P) [47,48] and Cl^-^ ions to produce a neutralized aqueous environment. The system was energy minimized using steepest descent algorithm [49] with a cut off energy value of 10.0kJ/mol for a maximum of 500 ps. Complex was then run through NVT and NPT equilibration for 100 ps each with position restraints applied for both protein and ligand. Finally, the MD simulation run of 250 ns was performed for each selected protein-ligand complex using leap frog integrator algorithm.

RMSD of ligands was calculated with respect to the backbone of protein to observe the extent of conformational changes in the position of ligand during the simulation period. This allows to observe the stability of ligand at the binding site of protein. RMSF of protein residues was also calculated to observe the residues that show high structural fluctuation throughout the simulation period. Additionally, to analyze the ligand protein interaction profile of the stable ligand-protein complexes, hydrogen bond analysis was performed. The hydrogen bonds were considered among atoms that formed an angle of <20o between donor hydrogen acceptor atoms at a distance < 3.0Å between donor acceptor atoms (hydrogen bonds plugin: Visual Molecular Dynamics 1.9.3) [50]. Then from all the detected hydrogen bonds, those that were persistent for > 0.5% of simulation time, were further investigated.

### ADMET analysis of compounds

The pkCSM online server [51] (http://biosig.unimelb.edu.au/pkcsm/prediction) was used for the the prediction of the absorption, distribution throughout the body, metabolism by accessory enzymes for excretion, removal as well as toxicity against common off targets in humans using SMILES of ligands as input.

## Results

### Homology modelling & MD simulation of Smad3

**Fig 1:**
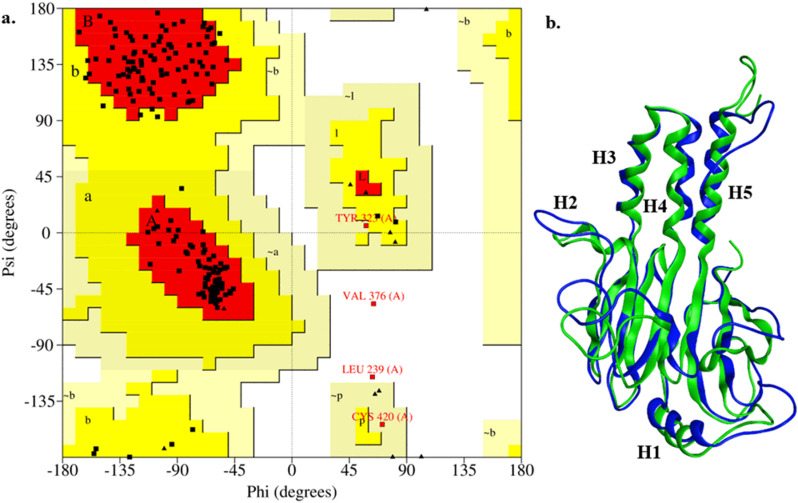
Evaluation of Smad3 homology model quality. **(a)** Ramachandran plot of model reveals that only 1.2% residues (Val376 and Leu239) have torsion angles in disallowed regions with an additional 1.2% (Tyr323 and Cys420) having torsion angles in generously allowed regions that is an indicator of high-quality of the structure. **(b)** Structure of Smad3 Mad Homology-2 (MH2) domain model before (green) and after (Blue) 50 ns MD simulation. Minimal structural changes at the core of model were observed (Root Mean Square Deviation = 5.09nm) while most of the changes are observed at the c- and n-terminus of structure. C-terminal residues after Gly414 to Ser424 were removed after simulation.

Homology model of human Smad3 MH2 domain was developed using the 1mjs and 1mks crystal structures of MH2 domain [35]. Based upon the higher resolution and the fact that it is in free state, 1mj2 (1.97Å) was used as a template for structural modeling of Smad3. Subsequently, the missing regions (residue 323–327 and 380–386) were modelled using 1mk2 (2.74Å) as template (S3 Fig). The best modelled structure was selected based upon the calculated Discrete Optimized Protein Energy (DOPE) score, highest number of residues having Psi and Phi angles within the favorable regions of Ramachandran plot (Fig. 1a) and ProsaWeb model parameters (S4 Fig)

MD simulation of structure revealed only a small deviation from the input structure during the 50 ns simulation time. It was observed from the Root Mean Square Deviation (RMSD) plot of protein backbone (Fig a in S5 Fig) that protein structure shows structural variation of 0.4nm in the initial 38 ns of simulation and then stabilizes. The structures of Smad3 MH2 domain model before and after MD simulation reveal an overall RMSD of 5.09nm (Fig 1b). While the Root Mean Square Fluctuation (RMSF) plot of structure indicates that majority of structural fluctuation is observed at the c-terminal loop region of protein (Fig b in S5 Fig).

### Dataset of inhibitors of Smad3

The preprocessed ligand dataset consisted of 33 compounds (S3 Table) that contained a complete or partial two-fold symmetry, and the inhibition potential greater than 50% (inhibition potential calculated by equation iii and iv) [37] was used for molecular docking and molecular dynamic simulations. The generated docking solutions were observed to be clustered in the groove present at the base of H2, and H4 helices (Fig b in S2 Fig). The ligand-protein interaction profiles of the binding solutions suggest that linker region of almost all compounds formed surface contacts with Arg367 at the base of H4 helix followed by Gln315 and Pro317 at the base of H2 helix that formed similar type of interactions (S6 Fig). Thus, it appears that these residues might act as anchoring point for ligands while the other regions of ligand formed interactions with residues at either side of this anchoring point (Fig b in S2 Fig). To further validate the role of these Arg367, Gln315 and Pro317, MD simulation of top pose (based upon binding energy calculated by MolDock score [42]) of each compound was performed.

### MD simulation of the docked complexes

The results of MD simulations suggest that only 13 out of 33 docked ligands form stable interactions within the binding site of FoxH1 protein (H2, H3 and H4 helix) (Table 1).

**Table 1:** Docked compounds that formed stable interactions with Smad3 MH2 domain at FoxH1 binding site.

Group-I	Group-II	Group-III
SM1	SM4	SM6
SM2	SM7	SM10
SM5	SM11	SM30
SM15	SM22	SM32
SM17		

**Fig 2:**
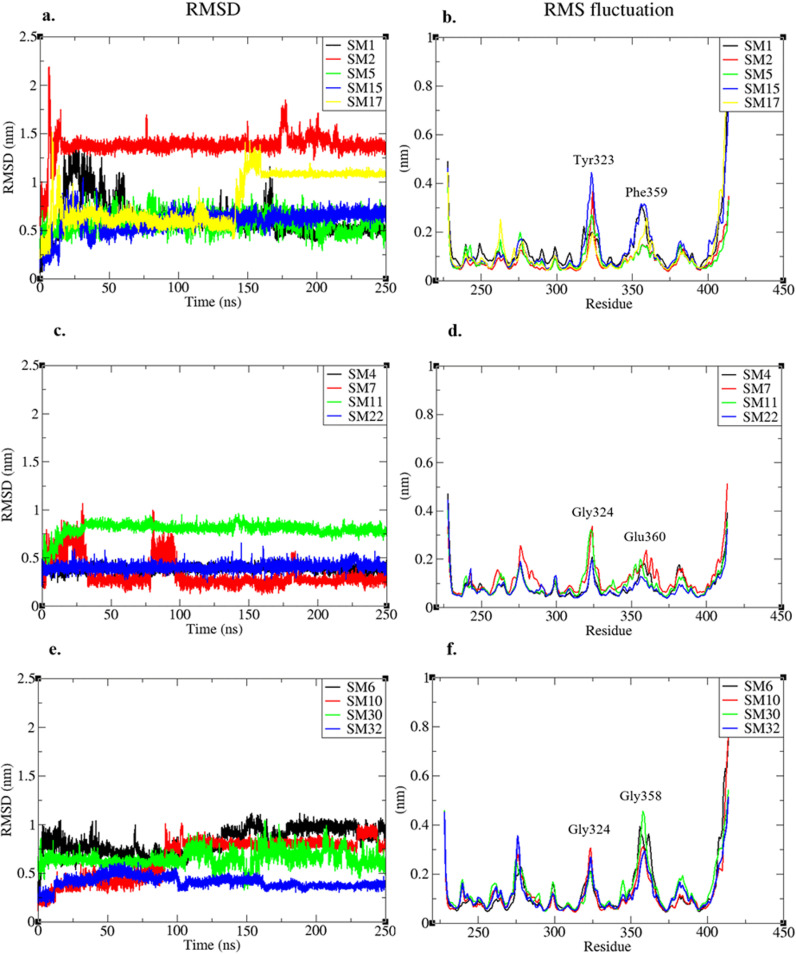
Root Mean Square Deviations (RMSD) and Root Mean Square Fluctuations (RMSF) of protein ligand complexes. Residues having highest local RMSF have been labelled. **(a)** RMSD of heavy atoms of group-I ligands least square fitted to protein as reference. **(b)** RMSF of proteins in complexed with group-I ligands. **(c)** RMSD of heavy atoms of group-II ligands least square fitted to protein as reference. **(d)** RMSF of proteins in complexed with group-II ligands. **(e)** RMSD of heavy atoms of group-III ligands least square fitted to protein as reference. **(f)** RMSF of proteins in complexed with group-III ligands.

The RMSF plot of protein in all complexes reveal (Figs 2b, 2d and 2f) that core protein residues remained stable except some amino acids upstream of the H2 helix (Tyr323 to Gly324 that form a small loop region) and some residues that join the H3 and H4 helix (Gly358 to Glu360).

#### RMSD and hydrogen bond interactions of group-I compounds stable at FoxH1 binding site.

Among the 13 stabilized complexes, the RMSD of ligands from group-I showed that these complexes achieved stability without any large change in binding position with respect to protein except for **SM2** and **SM17** (Fig 2a). A change in binding conformation was observed in **SM2** near the very start of MD simulation where ligand interactions with protein were slightly adjusted. The direct hydrogen bond of ligand with Arg367 observed in the molecular docking was transferred to a water-mediated hydrogen bond, and two new hydrogen bonds were formed with Asn320 and Gln315. In the case of **SM17**, no hydrogen bond was observed after the docking. However, a change in conformation after 160 ns simulation resulted in hydrogen bond formation with Gln364, Gln315 and Val388. The **SM5** showed a relatively flexible interactions with fluctuations of 0.58nm from 0.36nm to 0.94nm. Table 2 entails the hydrogen bond interactions of group-I ligands before and after MD simulation. These interactions suggest that after molecular docking, **SM1** and **SM2** from group I interacted with Arg367 and Asn320 while others did not form any hydrogen bond and were only docked based upon their structural fitness in this binding site. However, after MD simulation, Gln315 and Gln364 were also observed as common interacting residues for group-I ligands in addition to Asn320 and Arg367.

**Table 2:** Highlights the residues of Smad3 in MH2 domain that formed hydrogen bonds with group I ligands in before and during the 250 ns of Molecular Dynamic (MD) simulation.

			Hydrogen bond before MD				Hydrogen bond after MD				
Compound	Molecular Weight	Inhibition Potential					Direct			Water mediated	
SM1	396.40	102.17	Arg367	Thr387	Val388	Gln386	Asn320	Gln321		Asn320	Gln321
SM2	256.35	102.14	Arg367				Asn320	Gln315		Arg367	
SM5	392.33	99.29	Asn320	Gln315			Ala328	Arg367		Thr320	
SM15	783.46	89.73					Gln364	Ser235	Ala361	Gln364	
SM17	451.48	83.11					Gln364	Gln315	Val388		

**Fig 3:**
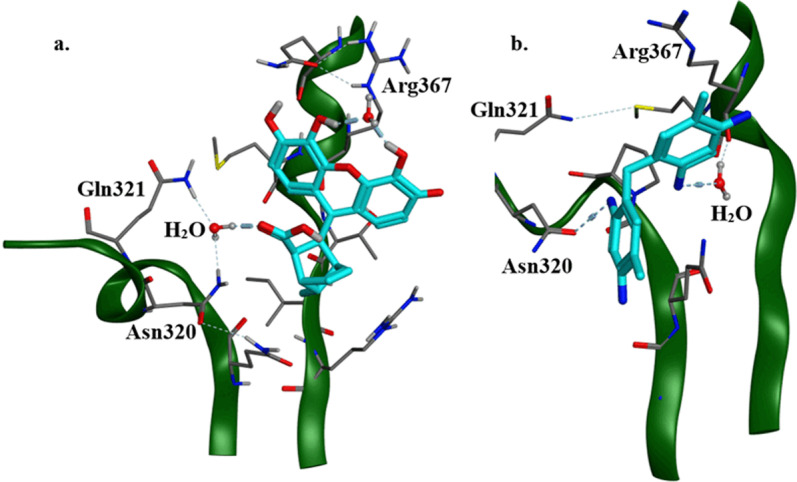
Interactions of two inhibitors with highest inhibition potential from group I with residues near H2 helix **a.** 3D interaction of SM1 with Smad3 Mad Homology 2 (MH2) domain after 250 ns of Molecular Dynamic simulation. **b.** 3D interactions of SM2 with Smad3 Mad Homology 2 (MH2) domain after 250 ns of Molecular Dynamic simulation.

The two compounds with highest inhibition potential (**SM1** = 102.17 and **SM2** = 102.14, Table 2), that also share strikingly high structural similarities showed interactions with Asn320. This interaction was not observed in any of the other stable conformations of compounds. The slightly higher inhibition potential of **SM1** over **SM2** can be contributed to that fact that the same hydrogen bonds, formed between **SM1** and protein are strengthened by water mediated hydrogen bonds (Table 2). Thus, it can be hypothesized that the high inhibition potential of **SM1** and **SM2** derives from their ability to form stable and consistent hydrogen bond with Asn320 (Fig 3)

#### RMSD and hydrogen bond interactions of group-II compounds stable at FoxH1 binding site.

**Fig 4:**
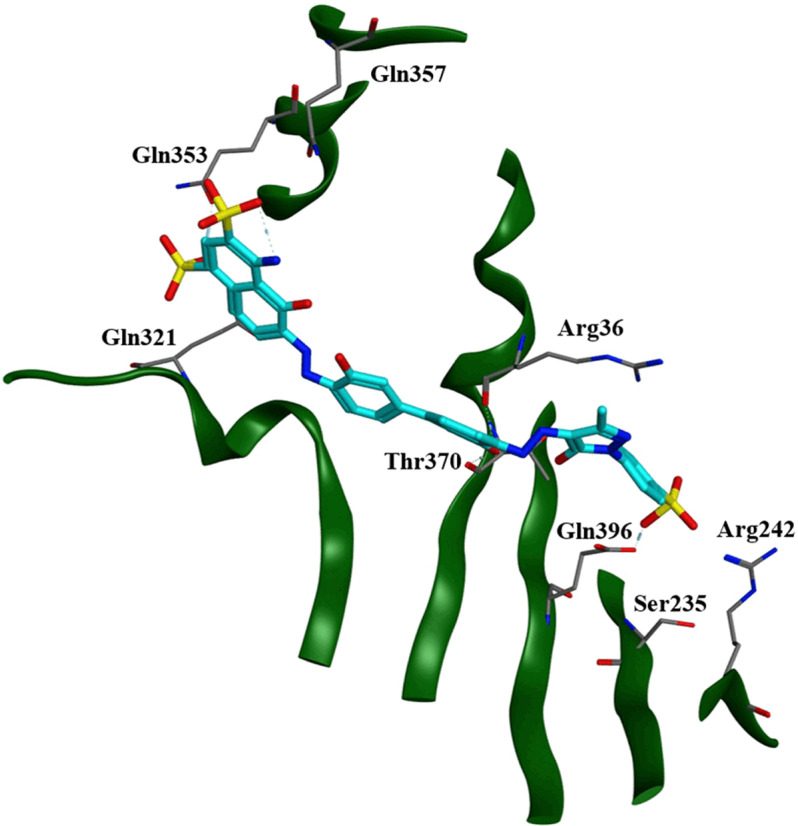
3D interaction of SM4 with Smad3 Mad Homology-2 (MH2) domain after 250ns of Molecular Dynamic (MD) simulation.

RMSD of four out of seven compounds from group-II also remained stable throughout the simulations with 0.2nm change in RMSD on average (Fig 2c). Further exploration of RMSD of **SM7**-Smad3 complex reveals that complex remained stable from 30 ns onwards within the binding site with RMSD change of 0.32nm from 0.11 to 0.43nm with frequent conformation disruptions at 80–100 ns and again at 160 ns. The hydrogen bond analysis of these complexes reveal that despite difference in their sizes and structure, these compounds formed similar hydrogen bond interactions (Table 3). While three out of four (SM4, SM11 and SM22) compounds formed hydrogen bonds with Arg367, Thr370 and Gln396 residues, two out of four compounds formed hydrogen bonds with Gln321 and Gln357 (Gln357 formed hydrogen bonds with SM4 and SM11 while Gln321 formed hydrogen bonds with SM4 and SM7). When compared to group-I ligands, it can be observed that although none of these compounds formed hydrogen bond with Arg367 before MD, this interaction formed by **SM5** and **SM2** (although via water molecule) was consistent in three out of four group-II compounds after MD simulation. **SM4** being the largest (molecular weight: 812) of all the compounds revealed highest number of hydrogen bond interactions with protein (Table 3). The interactions of **SM4** at the end of MD simulation can be observed in Fig 4.

**Table 3:** Highlights the residues of Smad3 and Mad Homology-2 domain that form hydrogen bonds with group-II ligands in before and during the 250 ns of Molecular Dynamic (MD) simulation.

Compound	Molecular Weight	Inhibition potential	Hydrogen bond before MD				Hydrogen bond after MD				
SM4	812.82	101.01	Pro317	Trp325	Asn240		Arg367	Thr370	Gln396	Gln321	Gln357
							Ser235	Gln353	Arg242		
SM7	302.19	97.61	Gln315	Thr370			Arg367	Thr370	Gln396	Gln321	
SM11	636.56	95.55	Gln315	Asn320	Val388	Ala328	Arg367	Thr370	Gln396	Gln364	Gln357
							Gln315				
SM22	382.63	69.89					Gln364				

#### RMSD and hydrogen bond interactions of group-III compounds stable at FoxH1 binding site.

Among the four compounds form group-III that stabilized at FoxH1 binding site, RMSD of **SM10** revealed rigorous fluctuations in conformation in the initial 50–100 ns but complex attained a stable conformation at 100 ns with RMSD of 0.13nm. Although **SM10** diverged from its position with an RMSD of 0.15nm at 230 ns, it regained its previous pose at 245 ns. RMSD of **SM6** revealed a pattern of convergence in structural configuration that stabilized around 170 ns with an average of 0.25nm (Fig 2e). The hydrogen bond analysis of these compounds suggests that **SM32** was only able to retain the hydrogen bond interaction with Ala328 while the interaction with Gln315 was lost during MD simulation. Meanwhile, all other stable compounds from group-III formed interactions with Gln364. This is followed by the Gln315 where two of three larger ligands (**SM10** & **SM30**) are observed to form stable hydrogen bonds (Table 4).

**Table 4:** Highlights the residues of Smad3 and Mad Homology-2 domain that form hydrogen bonds with group-III ligands before and during the 250 ns of Molecular Dynamic (MD) simulation.

			Hydrogen bond before MD					Hydrogen bond after MD	
Compound	Molecular Weight	Inhibition potential			Direct			Water mediated	
SM6	628.02	98.67			Gln364	Gln357	Ser354		
SM10	451.48	95.66	Glu396		Gln364	Ala328	Gln315		
SM30	484.60	55.42	Gln315		Gln364	Ala328		Asn320	Gln315
SM32	316.40	52.18	Gln315	Ala328	Ala328				

#### RMSD of compounds that detached from FoxH1 binding site in MD simulation.

In comparison, complexes that were not stabilized revealed diverse RMSD patterns (S7 Fig). Among these complexes **SM3**, **SM9**, **SM20**, **SM25** and **SM27** from group-I were not stabilized. Compared to the other highly potent inhibitors, **SM3** lacks the chemical groups capable of forming hydrogen bonds. This could be the cause of its instability at this binding site. Although **SM25** and **SM9** appeared to be stable in the initial 176 ns and 100 ns respectively, both of them detached from the binding site (Fig a in S7 Fig). The bulkier volume of additional ringed structure of **SM9** might be the cause of this disruption at this binding site. Additionally, with low inhibition potential of **SM25** (62%) and **SM27** (59%) we could argue that these compounds might affect the binding potential of Smad3 to FoxH1 through some other binding site.

**SM18, SM23** and **SM24** from group-II were considered unstable at H2, H3 and H4 helix binding site of Smad3 MH2 domain. This could be attributed to distant hydrogen bond donors in **SM18, SM23** and **SM24**. **SM18** appeared to be stable till 135 ns when it appeared to start detaching from the site (Fig a in S7 Fig), while **SM23** and **SM24** reattached themselves near the truncated N terminal of MH2 domain (Fig g in S7 Fig).

12 ligands (**SM8**, **SM12**, **SM13**, **SM14**, **SM16**, **SM19**, **SM21**, **SM26**, **SM28**, **SM29**, **SM31** and **SM33**) from group-III detached from the H2, H3 and H4 groove of MH2 domain. However, some of the compounds did form stable interactions with other sites of MH2 domain. For example, **SM14** stabilized at the truncated c-terminus of Smad3 structure. **SM16**, although stable for initial 130 ns, started detaching from the site (Fig c in S7 Fig). **SM29** stabilized at the truncated n-terminus of MH2 domain. Other than these, no complexes (Fig g in S7 Fig) produced any stable conformations after 250 ns of simulation. It is worth mentioning here that majority of group-III ligands were unable to produce stable complexes at Smad3 MH2 domain and FoxH1 binding interface. So, it can be hypothesized that dimeric compounds having a linker region of greater than 2 bond distances bind with Smad3 protein at sites having distant hydrophobic patches.

#### Novel binding site for SM8 and SM19 from group-III.

**Fig 5:**
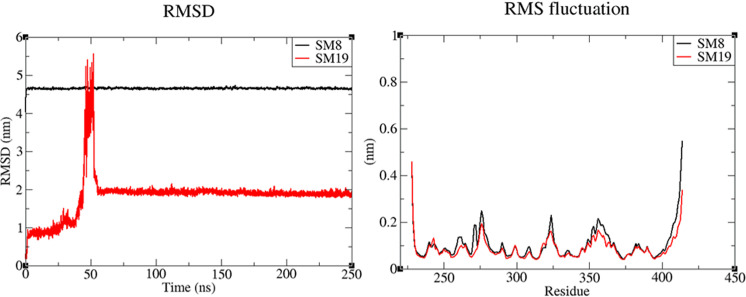
Root Mean Square Deviation (RMSD) and Root Mean Square Fluctuation (RMSF) for complexes with ligand stable at novel binding sites. **(a)** RMSD of SM8 and SM19 ligands that formed stable complexes around Tyr323. **(b)** RMSF of Mad Homology-2 (MH2) domain residues complexed with SM8 and SM19.

**Fig 6:**
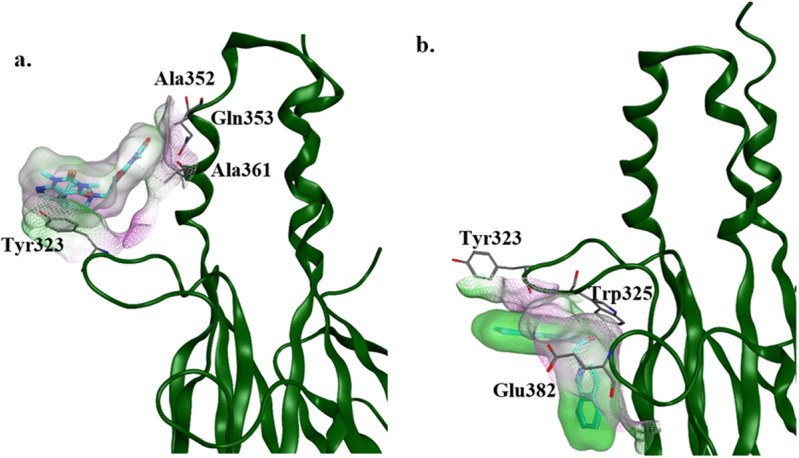
Ligand protein interactions of SM8 and SM19. (a) **SM8** stable binding pose after 250 ns of Molecular Dynamic (MD) simulation. **(b) SM19** stable binding pose after 250 ns of MD simulation. The green color represents hydrophobic surface while pink color represent hydrophilic surface area.

Among these complexes, the behavior of **SM8** and **SM19** is interesting. **SM8** stabilized at the start of MD simulation between Tyr323 near H2 helix and Ala349 in H4 helix (Figs 5 and 6). This complex is stabilized by the hydrophobic complementarity among the closed rings of ligand and Tyr323 side chain (Fig 6a). This site is known as the binding site where Ski protein interacts with Smad3 protein [35]. On the other hand, SM19 stabilized after 55 ns of MD simulation between Tyr323 and Tyr383 having similar hydrophobic complementarity with the side chains of Tyr residues (Fig 6b).

### Smad3-MH2 domain residues frequently contributing to stable hydrogen bond interactions

**Fig 7:**
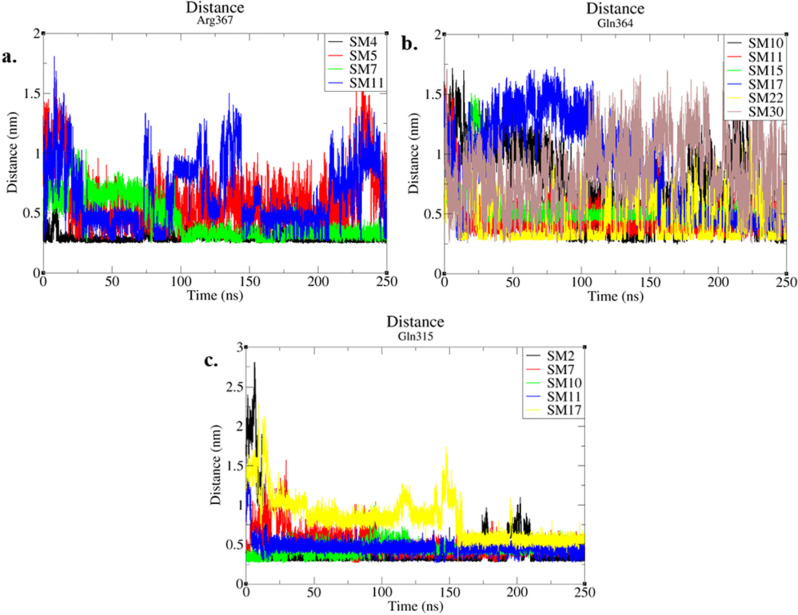
The distance of donor acceptor atoms for most commonly hydrogen bonds forming residues in this study. The stability of hydrogen bonds can be observed by the fluctuations in the distance of donor acceptor atoms. **(a)** The hydrogen bonds formed by the Arg367 with SM4 and SM7 are very stable. While the ones formed with SM5 and SM11 are relatively less stable **(b)** A similar pattern is observed with Gln364 where the hydrogen bonds formed with SM30 is not that much stable when compared to other ligands. **(c)** In case of Gln315, all hydrogen bonds look very stable with the exception of SM17 where the distance among donor acceptor atoms is a bit higher compared to other ligands.

Among all 13 stabilized complexes Gln364, Gln315 and Arg367 formed the highest number of hydrogen bonds with ligands at FoxH1 binding site. Arg367 formed hydrogen bonds with three out of five stable group-II ligands in addition to a water mediated hydrogen bond with **SM1** ligand from group I (Fig 7a). Gln364 was observed to form hydrogen bonds with **SM15** and **SM17** in group-I ligands as well as with all larger stable group III ligands (Fig 7b). While Gln315 was quite frequently observed in the docking solutions of all 33 compounds investigated here, it was later observed in a few stable poses from each ligand group (Fig 7c). Nevertheless, hydrogen bonds of **SM11**, **SM17** and **SM30** with the above-mentioned residues are not very stable. But the synergistic effect of these short living hydrogen bonds was strong enough to keep the ligand within binding site.

### ADMET analysis of compounds

ADMET properties can provide valuable insights into the physiochemical properties of a compounds that are important for a potential drug candidate [52–54]. We have tested the ADMET properties all active dimeric inhibitors of Smad3 investigated in this study to observe their potential as drug candidate in ASCVD. In this regard, we tested the intestinal absorption, water solubility, and bioactivities against the toxicity targets including hERG and CYP isoforms, and AMES-toxicity of the compounds (S5 Table). The ADMET analysis reveals that this dataset (PubChem ID: 630) contains a diverse set of compounds with respect to their combined tested ADMET properties.

In case of water solubility, the optimal calculated logS of a compounds should be close to or greater than 0. Whereas, compounds with logS less than −4 can be classified as insoluble in water [55]. The logS of the active dimeric compounds tested in this study range from −2.86 in **SM18** (slightly water solube) to −7.96 in **SM19** (water insoluble).

The predicted intestinal absorption of a molecule indicates the bioavailability of candidate molecule that can reach the bloodstream through oral intake. Our dataset contained active Smad3 inhibitors with intestinal absorption ranging from 0.06% (**SM4**) to 100% (**SM3**).

Bioactivities of compounds against CYP isoforms depict the potential of molecule to inhibit xenobiotic metabolism in human body [56]. We tested the toxicities of compounds against 7 isoforms of CYP as well as two isoforms of hERG channels and AMES-test. Overall, **SM8**, **SM11**, **SM25** and **SM31** produced no toxicity against any of 10 targets while **SM3** was predicted to be toxic against 7 out of 10 tested targets. The detailed ADMET properties of all 33 dimeric active inhibitors of Smad3-FoxH1 binding are listed in S5 Table.

Overall, **SM8**, **SM11**, **SM25** and **SM31** revealed no toxicity with **SM8** and **SM11** having high inhibition potential (**SM8** = 97.61% and **SM11** = 95.55%) and low intestinal absorption while **SM25** and **SM31** having higher intestinal absorption and lower potency (**SM25** = 62.40% and **SM31** = 54.69%).

## Discussion

In this study, we have investigated the binding hypothesis of Smad3 inhibitors. Most of the previous studies regarding exploration of Smad3 inhibitors focused on small number of substances revealing only one or two active compounds [57–60]. Only two large scale studies were conducted against Smad3. Herein, the data from one of those studies (PubChem ID:630) [37], that focused on the inhibition of Smad3-FoxH1 binding, was used. We have applied the MolDock algorithm [42] to generate initial binding conformations for these inhibitors and validated these bindings by MD simulations of 250 ns using AMBER99 ILDN [33] force field in GROMACS 2019.6 [43]. To further investigate the interactions responsible for stable binding conformations, we analyzed the hydrogen bonds among compounds and Smad3 protein for their consistency during the MD simulation.

Our findings suggest that the selected dataset of the inhibitors of Smad3 showed diverse binding pattern. This statement can be supported by the diversity of these inhibitors in size as well as in number of hydrogen bond forming moieties. Additionally, Smad3 can also be observed to form a diverse array of binding interactions with modulatory proteins. These binding partners include auxiliary protein SARA, inhibitory proteins, i.e., Evi, Sno, Ski, Smad6, Smad7, c-myc and transcriptional factors, i.e., FoxH1, c-jun, c-Fos, SIP1/ZEB2 [35,36,61–65]. For most of the Smad3 binding partners, the interaction patterns between Smad3 and binding protein are not well characterized. Some of the better studied interactions include the c-terminal interactions of Smad3 with SARA and TGFβ receptor complex, and the interactions of Smad proteins with Ski and FoxH1 [35,36]. In these studies, region between H3, H4 and H2 helices has been reported to interact with FoxH1 protein [36]. While the β-sandwich and H5-helix regions of MH2 domain have been identified as binding sites for SARA protein [35]. Suggesting that MH2 domain of Smad3 is capable of binding with various proteins through different binding sites. And while we were able to characterize the interactions of 13 out of 33 compounds at FoxH1 binding site, the remaining compounds (like SM9 and SM18) could bind at different regions of Smad3 to produce their inhibitory effects.

The 13 compounds that formed stable interactions at FoxH1 binding site were grouped into three classes based upon the length of linker region (linker region of two bond distance: group-I, linker region of one bond distance: group-II, remaining compounds with variable linker region: group-III) joining the aromatic structures. Although the length of the linker region allows molecules to form different pattern of interactions with the binding site, no significant difference of activities was observed based upon this feature. Only in **SM15** and **SM30**, the linker region was observed to directly form hydrogen bonds with residues of Smad3 (S8 Fig). The most potent Smad3 inhibitors from the previous study (PubChem ID: 630) [37], **SM1** and **SM2** have a linker region of two bond distance. The MD simulation of these revealed the ability to form direct stable hydrogen bond with Ans320. Although 13 compounds stabilized at binding site that includes Asn320, no other compound was able to form this interaction. Thus, it can be hypothesized that if a compound can find a shape complementarity to Smad3 binding site around Asn320 closer to the H2 helix and is able to form stable hydrogen bond with Asn320, it could efficiently inhibit Smad3-FoxH1 binding. Among the compounds with highest inhibition potential, **SM3** does not contain the -OH or -NH groups and thus was not observed to form hydrogen bonds at the Smad3 FoxH1 binding site. It is possible that it might interact with Smad3 via hydrophobic interactions as observed with **SM8** and **SM19**. Compounds with linker region of one bond distance show slightly less activity against Smad3. While compounds with distance greater than two bond length among ringed structures were observed to be least stable at FoxH1 binding site. The stable hydrophobic interactions of **SM8** and **SM19** suggest that compounds with linker region greater than two bond length joining the hydrophobic ring structures might form interactions with distant hydrophobic patches of Smad3.

Among the 13 compounds (**SM1, SM2, SM4, SM5, SM6, SM7, SM10, SM11, SM15, SM17, SM22, SM30** and **SM32**) that were found to stably bind at the FoxH1 binding site, only three compounds (**SM22, SM30** and **SM32**) had the inhibition potential of less than 80%. For **SM22** (inhibition potential = 69.89%), only a single stable hydrogen bond with Gln364 was observed. This interaction although allowed the compound to retain at the binding site, the location of Gln364 (H4 helix, S8 Fig) is a bit distant from the H2, H3 and H4 helix junction. **SM30** (inhibition potential = 55.42%) formed a very weak hydrogen bond with Gln364 along with the hydrogen bond with Ala328 and water mediated hydrogen bonds with Asn320 and Gln321 allowed this compound to remain attached. While **SM32** (inhibition potential = 52.18%) remain attached to Smad3 by only a single hydrogen bond interaction with Ala328. These interactions suggest that compounds that are capable to interact with Smad3 at FoxH1 binding site with weak or single direct hydrogen bond that are away from the H2, H3 and H4 helix junction have lower inhibitory activity.

Regardless of the length of linker regions, larger ligands (molecular weight > 625) **SM4**, **SM11** and **SM15** were observed to form similar interactions with the Smad3 by forming hydrogen bonds with Arg367, Thr370 and Gln396. Further exploration of these interactions reveals that **SM4** and **SM11** contain abundant hydrogen bond donors while **SM15** contains hydrogen bond donors that can be mapped to similar distances in 3D conformations. Suggesting that presence of hydrogen bond capable moieties at distance of five to seven bond length in large hydrophobic compounds is a favorable feature for Smad3 inhibitors. In addition to that, the binding pattern of **SM9** and **SM18** reveal that Smad3 region around Tyr323 can be considered as a binding site for Smad3 FoxH1 inhibitors. As Tyr323 also interacts with FoxH1 near the C terminal [36].The ADMET analysis of the dataset reveals **SM8**, **SM11**, **SM25** and **SM31** the best possible candidates for further *in vitro* and *in vivo* investigations based upon their low toxicity to CYP and hERG as well as low AMES-toxicity.

Regardless of the extensive and careful analysis taken at each step of the study, it is pertinent to mention that molecular docking and molecular dynamic simulation protocols tend to provide a virtual environment that can mimic the biological environment with minimal number of variables to reduce the computational load. And may not reflect the *in vitro* and *in vivo* systems completely. Thus, the development of molecules as potential drugs based upon computational models warrant *in vitro* and *in vitro* investigations [66,67].

## Conclusion

Literature evidences point out strongly toward the importance of Smad3 protein in coronary heart diseases. Although many studies have been done to discover compounds for modulation of Smad3 protein, discovery of Smad3 modulators still remains a challenge due to unknown mechanism of action. In this study we used the data of previous studies to generate a consensus binding hypothesis for Smad3 FoxH1 binding inhibitors. MolDock docking algorithm [42] was used to generate initial binding poses for 33 compounds with perfect or partial two fold symmetry. The stability of these complexes was validated through 250 ns MD simulations performed in an aqueous environment. Our results revealed that a some of the compounds in our analysis form stable interactions at Smad3 FoxH1 binding interface. Among these interactions, Asn320 appears to be an important anchor for hydrogen bond-based interactions with compounds having highest inhibition potential. Molecules that have shape and electrostatic complementarity allowing them to bind at the base of H2 helix with hydrogen bond donor groups that allow interaction with Asn320 can act as potent inhibitors of Smad3 protein. On the other hand, compounds having distant hydrogen bond forming groups attached to them at a distance of five to seven bond length can interact with Arg367, Gln364 and Gln396 in Smad3. This allows their stable retention at the H2, H3 AND H4 helix junction allowing inhibition of Smad3 FoxH1 binding. In addition to that, two compounds formed stable hydrophobic interactions around Tyr323 suggesting a novel Smad3 modulation site. The binding pattern of these compounds can provide important insights for development of potent Smad3 inhibitors with less toxic effects.

## Supporting information

S1 Fig Overview of the ligand data preprocessing. In 1^st^ step, 16 available were evaluated based upon the number of compounds being tested (Table. 2.) Most of these studies were observed to be limited to a low number of substances that inhibit the Transforming Growth Factor β/Smad3 pathway either through unknown mechanisms or by inhibiting the TGFβ-Receptor. In 2^nd^ step, the active compounds from the selected study (Pubchem ID: 630) were extracted. This data was then preprocessed as 3^rd^ step by i) removing possible artifacts reported in the experiment ii) compounds with no Compound ID removed iii) small fragment with molecular weight below 200 removed iv) racemic compounds removed v) only structures with dimeric symmetry retained. Compounds that passed through each of these filters are mentioned in this step. In 4^th^ step these 33 dimers were docked to Smad3 at Smad3-FoxH1 binding interface using MolDock algorithm.
(TIFF)

S2 Fig Molecular Docking of selected ligands. (a) Binding cavity selected for docking based upon literature evidences of interaction of this region at the base of H3, H4 and H2 helices as well as detection of this region as the largest binding cavity by MoleGro Virtual Docker 6.0. (b) All the docked poses generated by MoleGro Virtual Docker 6.0 [5] clustered at the base of H3, H4, H5 and H2 helices indicating possibility of a range of binding interactions.
(TIFF)

S3 Fig Sequence alignment of Smad3-MH2 domain query sequence for model and available residues in 1mjs crystal structure. The missing residues (323–327 and 380–386) were modelled using 1mk2 crystal structure.
(TIFF)

S4 Fig Quality parameters of Smad3 model. (a) Z-score of Smad3-MH2 domain model calculated by Prosa web server (https://prosa.services.came.sbg.ac.at/prosa.php) compared to the XRD (light blue background) and NMR (dark blue background) structures in PDB reveal that model quality is comparable to those present in PDB. (b) Energies of residues as averaged over a window of 10 (light green line) and 40 (dark green line) reveal that structure is quite stable with only a few spikes moving towards positive energy when energy in averaged over sliding window of 10 residues.
(TIFF)

S5 Fig MD simulation of prepared Smad3 model. (a) The Root Mean Squared Deviation (RMSD) of protein structure with reference to the initial structure reveals that the structure observes minor change over the course of 0−38 ns of simulation with fluctuations of 0.4nm from 0.2–0.6nm and then stabilizes. (b) Root Mean Square Fluctuations (RMSF) of residues reveal that most of the structural variations were observed at the C-terminal residues.
(TIFF)

S6 Fig Protein Ligand Interaction Fingerprints (PLIF) of docked complexes generated by Molegro Virtual Docker 6.0. Most of the docked ligands formed surface contact interactions with Arg367 present at the base of H4 helix followed by Gln315 and Pro317 at the base of H2 helix.
(TIFF)

S7 Fig MD simulation of ligands unstable at FoxH1 binding site. (a and c) Root Mean Square Deviation (RMSD) of compounds that are unable to stabilize within the binding site of FoxH1 in Smad3-MH2 domain. (b and d) Roor Mean Square Fluctuations (RMSF) of Smad3-MH2 2 domain while complexed with these compounds (e) RMSD of compounds that detached from the binding site of FoxH1 in Smad3-MH2 domain. (f) RMSF of Smad3 MH2 domain while complexed with compounds in e. (g) Compounds that attached to either N- or C-terminal truncated regions of Smad3-MH2 domain (h). RMSF of Smad3-MH2 domain while complexed with compounds in g.
(TIFF)

S8 Fig Hydrogen bond interactions of SM15 and SM30. (a) Hydrogen bond interactions of SM15 and Smad3 protein observed during Molecular Dynamic simulation highlighted along with their distances in the final frame of simulation. (b) Hydrogen bond interactions of SM30 and Smad3 protein observed during Molecular Dynamic simulation highlighted along with their distances in the final frame of simulation.
(TIFF)

S1 Table List of available structures of Smad3-Mad Homology-2 domain in Protein Data Bank. (PDF)

S2 Table List of available datasets for Transforming Growth Factor (TGF) β inhibitors available on PubChem. (PDF)

S3 Table List of 33 dimeric active Smad3 FoxH1 binding inhibitors. The activity threshold is set to 50% inhibition or above. Compounds have been ranked according to their activity in a descending order.
(PDF)

S4 Table List of compounds grouped into three categories. Group-I: Ligands with ringed structures around the plane of symmetry at a distance of two-bond length. Group-II: Ligands with ringed structures around the plane of symmetry at a distance of one-bond length. Group-III: Ligands with ringed structures around the plane of symmetry at a distance of three or more bond length.
(PDF)

S5 Table ADMET properties of the dataset. Revealing the potential of these compounds as drug candidates for ASCVD. The logS of compound should be between close to 0 (0 = water soluble, 0 to −1 = somewhat water soluble, −1 to −4 = slightly water soluble, less than −4 = water insoluble). Intestinal absorption is the percentage of drug absorbed through intestine. Metabolism reveal that out of seven tested CYP isoforms, how many isoforms are inhibited by the tested compound (ideally should be zero). AMES toxicity predicts weather the compound has carcinogenic/mutagenic potential or not. hERG toxicity again reveal the number of hERG channels (out of two tested) being inhibited by the compound.
(PDF)

S1 MD The input files used for MD simulation of the protein-ligand complexes. (ZIP)
