# Supplementary figures and images for: Probing the binding hypothesis of Smad3 modulators by molecular dynamic simulations for Atherosclerosis Cardiovascular Disease (ASCVD)
Source: PLoS One. 2025 Jun 4;20(6):e0324677. doi: 10.1371/journal.pone.0324677 (PMC12136405; doi:10.1371/journal.pone.0324677)

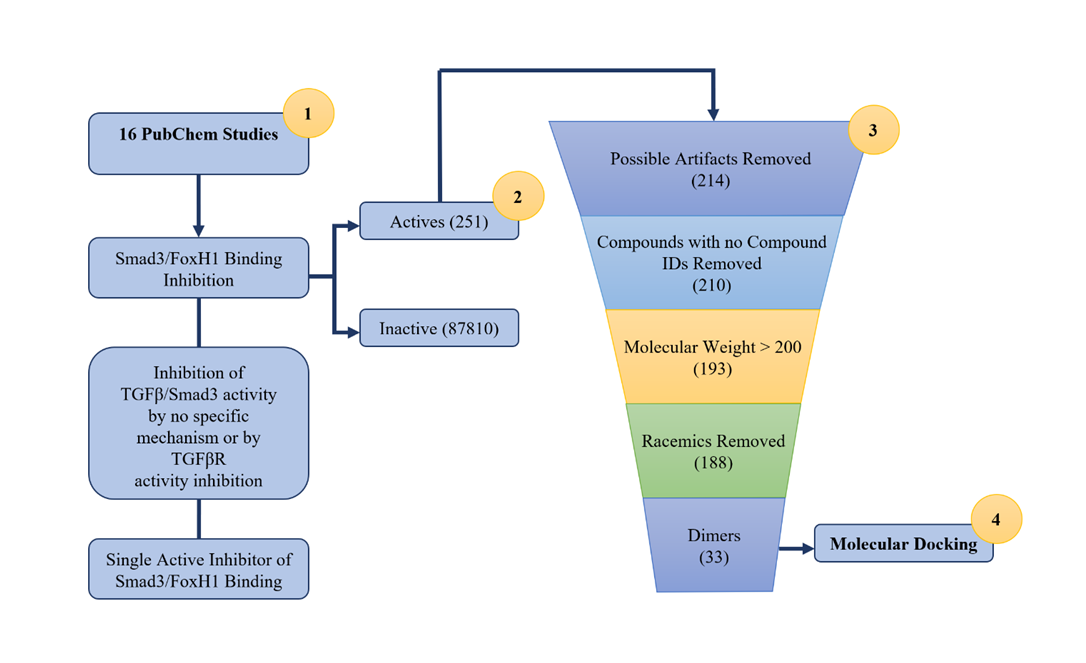

Supplement: S1 Fig — In 1st step, 16 available were evaluated based upon the number of compounds being tested (Table. 2.) Most of these studies were observed to be limited to a low number of substances that inhibit the Transforming Growth Factor β/Smad3 pathway either through unknown mechanisms or by inhibiting the TGFβ-Receptor. In 2nd step, the active compounds from the selected study (Pubchem ID: 630) were extracted. This data was then preprocessed as 3rd step by i) removing possible artifacts reported in the experiment ii) compounds with no Compound ID removed iii) small fragment with molecular weight below 200 removed iv) racemic compounds removed v) only structures with dimeric symmetry retained. Compounds that passed through each of these filters are mentioned in this step. In 4th step these 33 dimers were docked to Smad3 at Smad3-FoxH1 binding interface using MolDock algorithm. (TIFF) [file pone.0324677.s001.tiff]

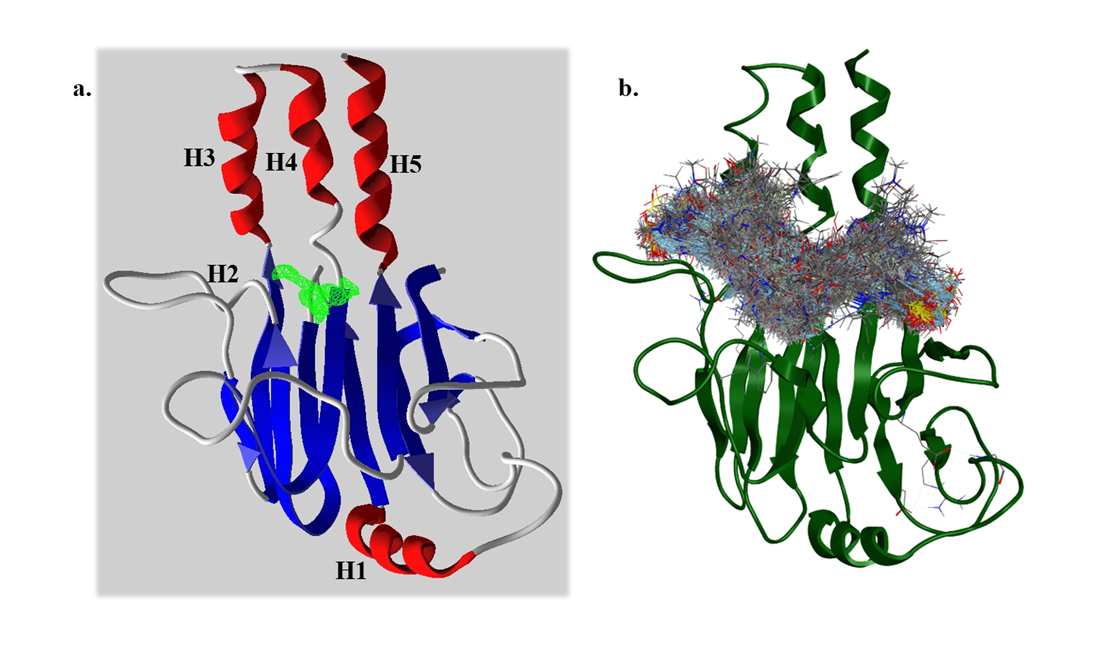

Supplement: S2 Fig — (a) Binding cavity selected for docking based upon literature evidences of interaction of this region at the base of H3, H4 and H2 helices as well as detection of this region as the largest binding cavity by MoleGro Virtual Docker 6.0. (b) All the docked poses generated by MoleGro Virtual Docker 6.0 [5] clustered at the base of H3, H4, H5 and H2 helices indicating possibility of a range of binding interactions. (TIFF) [file pone.0324677.s002.tiff]

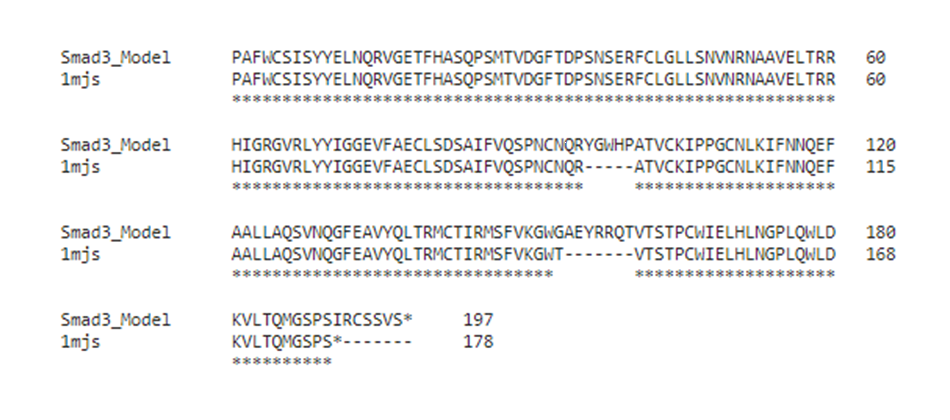

Supplement: S3 Fig — The missing residues (323–327 and 380–386) were modelled using 1mk2 crystal structure. (TIFF) [file pone.0324677.s003.tiff]

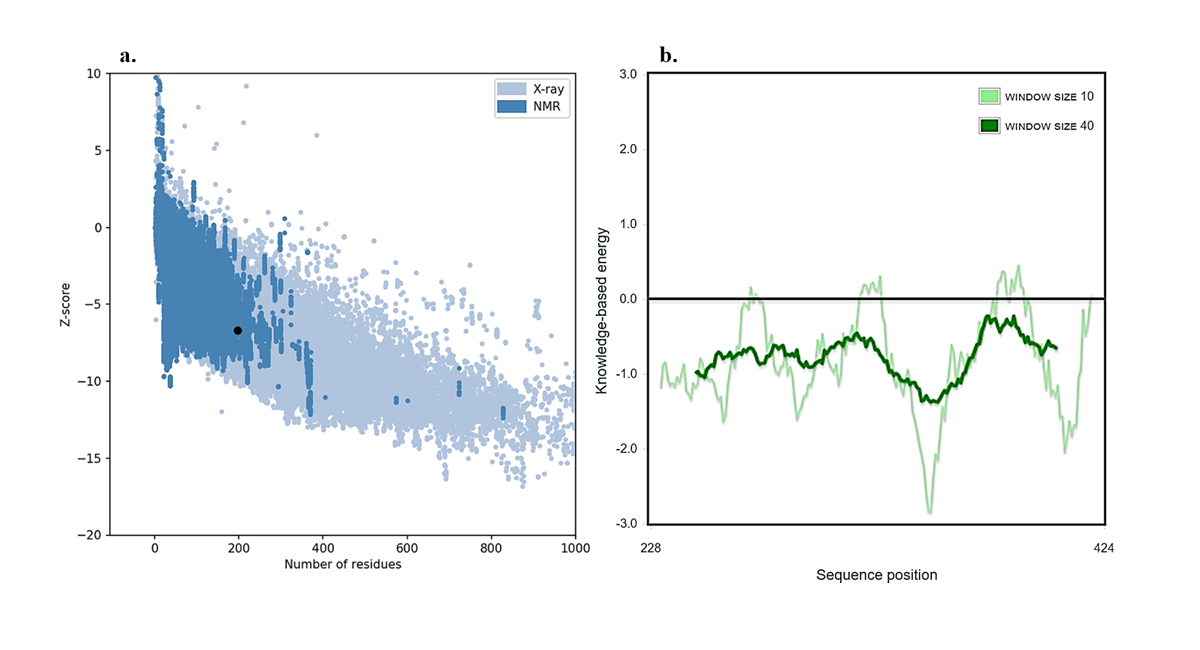

Supplement: S4 Fig — (a) Z-score of Smad3-MH2 domain model calculated by Prosa web server (https://prosa.services.came.sbg.ac.at/prosa.php) compared to the XRD (light blue background) and NMR (dark blue background) structures in PDB reveal that model quality is comparable to those present in PDB. (b) Energies of residues as averaged over a window of 10 (light green line) and 40 (dark green line) reveal that structure is quite stable with only a few spikes moving towards positive energy when energy in averaged over sliding window of 10 residues. (TIFF) [file pone.0324677.s004.tiff]

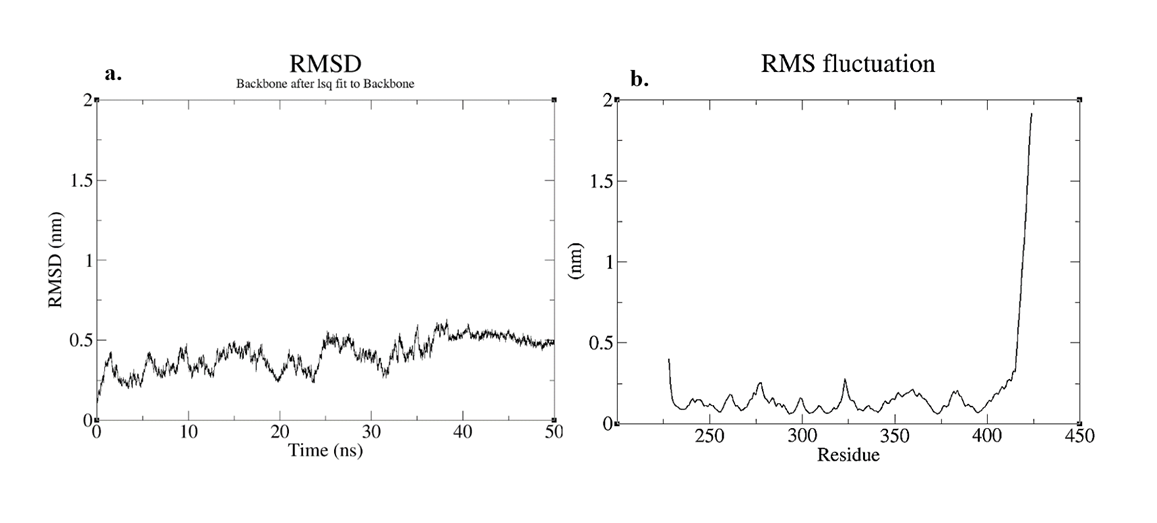

Supplement: S5 Fig — (a) The Root Mean Squared Deviation (RMSD) of protein structure with reference to the initial structure reveals that the structure observes minor change over the course of 0−38 ns of simulation with fluctuations of 0.4nm from 0.2–0.6nm and then stabilizes. (b) Root Mean Square Fluctuations (RMSF) of residues reveal that most of the structural variations were observed at the C-terminal residues. (TIFF) [file pone.0324677.s005.tiff]

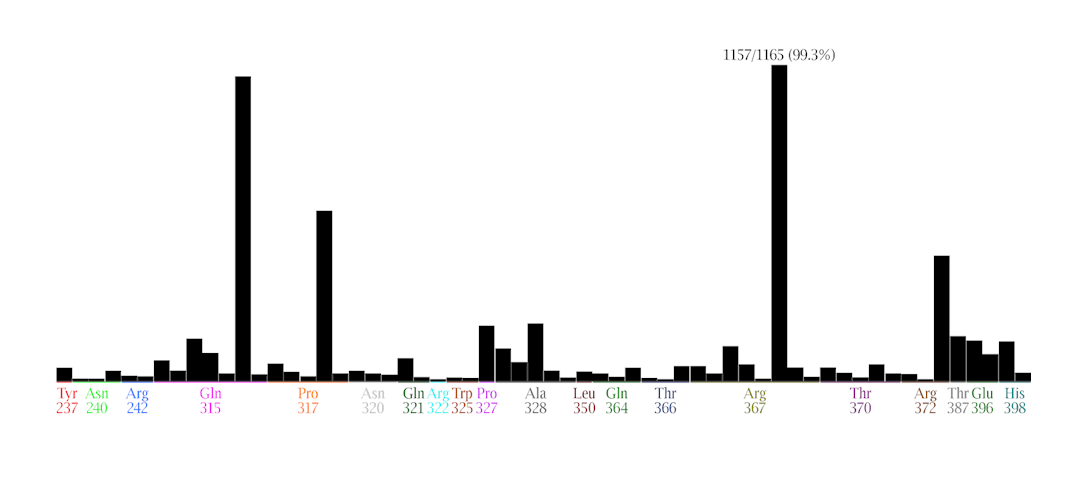

Supplement: S6 Fig — Most of the docked ligands formed surface contact interactions with Arg367 present at the base of H4 helix followed by Gln315 and Pro317 at the base of H2 helix. (TIFF) [file pone.0324677.s006.tiff]

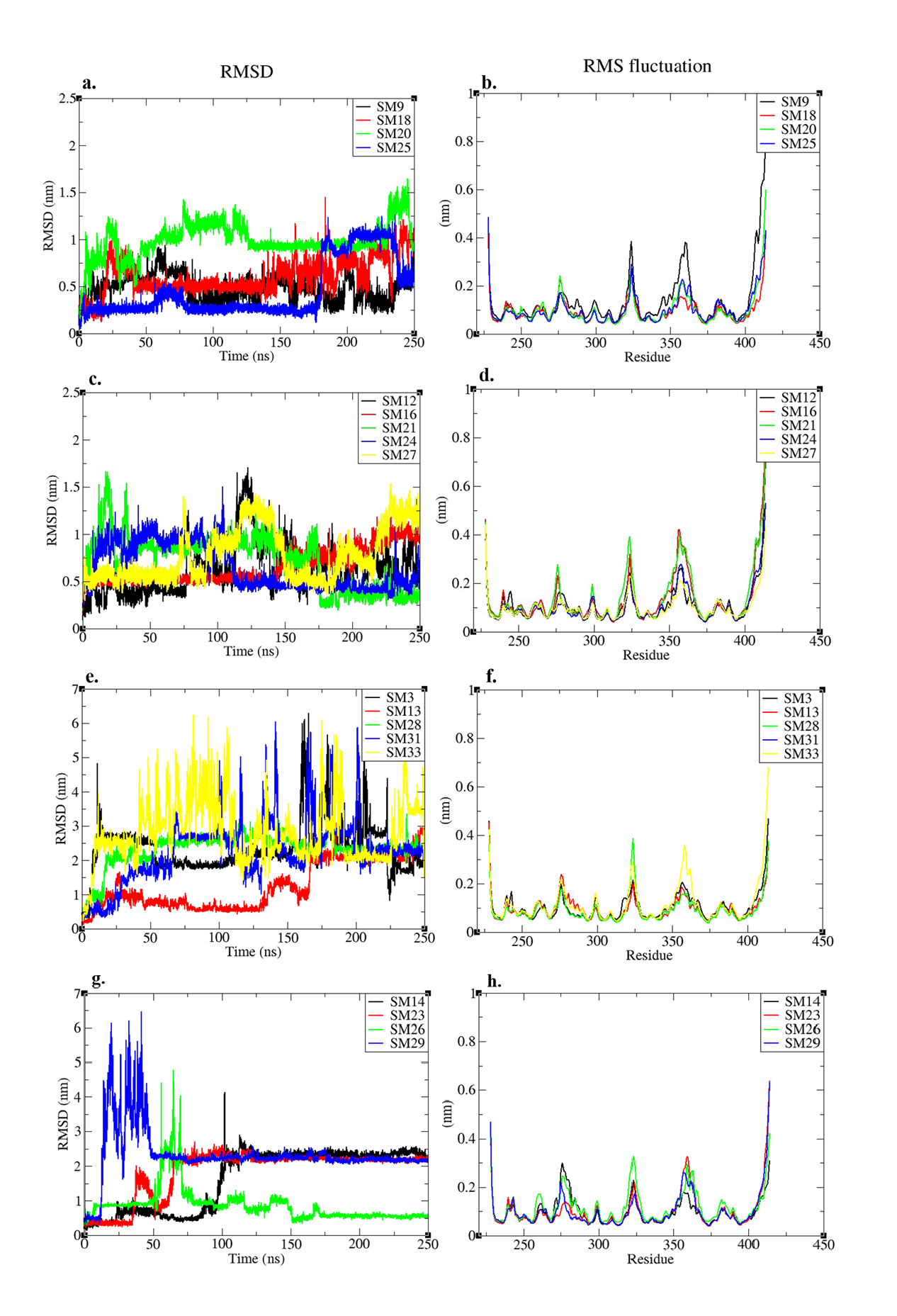

Supplement: S7 Fig — (a and c) Root Mean Square Deviation (RMSD) of compounds that are unable to stabilize within the binding site of FoxH1 in Smad3-MH2 domain. (b and d) Roor Mean Square Fluctuations (RMSF) of Smad3-MH2 2 domain while complexed with these compounds (e) RMSD of compounds that detached from the binding site of FoxH1 in Smad3-MH2 domain. (f) RMSF of Smad3 MH2 domain while complexed with compounds in e. (g) Compounds that attached to either N- or C-terminal truncated regions of Smad3-MH2 domain (h). RMSF of Smad3-MH2 domain while complexed with compounds in g. (TIFF) [file pone.0324677.s007.tiff]

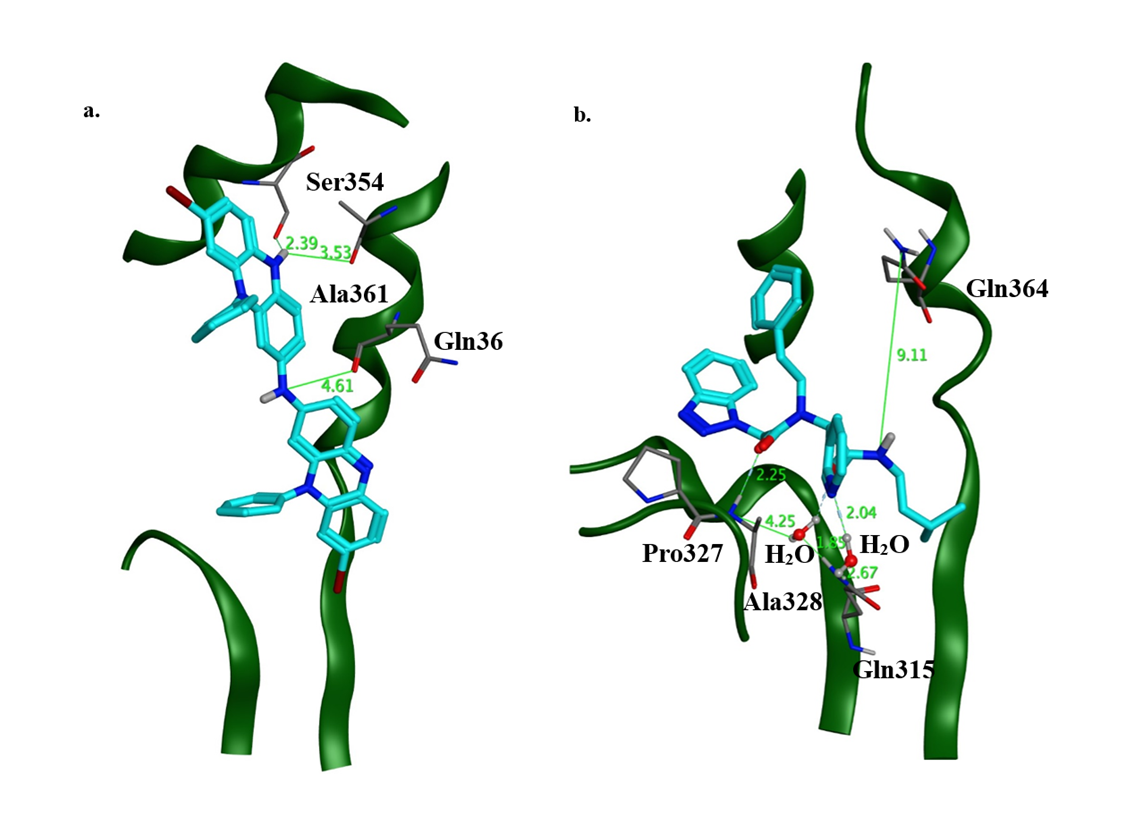

Supplement: S8 Fig — (a) Hydrogen bond interactions of SM15 and Smad3 protein observed during Molecular Dynamic simulation highlighted along with their distances in the final frame of simulation. (b) Hydrogen bond interactions of SM30 and Smad3 protein observed during Molecular Dynamic simulation highlighted along with their distances in the final frame of simulation. (TIFF) [file pone.0324677.s008.tiff]
