# Supplementary material for: Probing the binding hypothesis of Smad3 modulators by molecular dynamic simulations for Atherosclerosis Cardiovascular Disease (ASCVD)
Source: PLoS One. 2025 Jun 4;20(6):e0324677. doi: 10.1371/journal.pone.0324677 (PMC12136405; doi:10.1371/journal.pone.0324677)
Supplement: S1 Table — (PDF) [file pone.0324677.s009.pdf]

S. No.	PDB ID	Conformation (Human)	Resolution (Å)	Year
1	1MJS	MH2 Domain	1.91	2002 [1]
7	5XOC	SMAD3-FoxH1 Complex	2.40	2017 [2]
8	1UF7	SMAD3-SMAD4 Phosphorylated Hetero-trimer	2.60	2004 [3]
9	1MK2	SMAD3 SBD Complex	2.74	2002 [1]

[1] Qin, Bin Y., et al. "Smad3 allostery links TGF-β receptor kinase activation to transcriptional control." Genes & development 16.15 (2002): 1950-1963.

[2] Martin-Malpartida, Pau, et al. "Structural basis for genome wide recognition of 5-bp GC motifs by SMAD transcription factors." Nature communications 8.1 (2017): 1-15.

[3] Chacko, Benoy M., et al. "Structural basis of heteromeric smad protein assembly in TGF-β signaling." Molecular cell 15.5 (2004): 813-823.
