# Supplementary material for: Probing the binding hypothesis of Smad3 modulators by molecular dynamic simulations for Atherosclerosis Cardiovascular Disease (ASCVD)
Source: PLoS One. 2025 Jun 4;20(6):e0324677. doi: 10.1371/journal.pone.0324677 (PMC12136405; doi:10.1371/journal.pone.0324677)
Supplement: S2 Table — (PDF) [file pone.0324677.s010.pdf]

S. No.	PubChem ID	Multiple studies in Pubchem (538,745)						Remarks
		Study	Assay	Actives	In-actives	In-conclusives	Total	
**1**	588855	qHTS for Inhibitors of TGF-b	HEPG2-cell lines CAGA-GFP Bioassay	4993	351208	51591	407798	CAGA-response based Green Fluorescent Protein signal as activity of Smad3
**2**	720536	qHTS for Inhibitors of TGF-b: CCL64 Cells Orthogonal Assay for Cherry Picks	CCL642-cell lines CAGA-GFP Bioassay	652	296	624	1572	Same Bioassay as of PID: 588855 but tested on different cell lines
**3**	1346924	qHTS assay to identify small molecule antagonists of the TGF-beta/Smad signaling pathway	SBE-bla HEK 293T cell lines - B-lactamase under the control of Smad Binding Element. FRET substrate used as reporter fluorescence	54	7469	2000	9523	Similar Bioassay as of PID: 588855 but Different Reporter Technique
**4**	1346859	qHTS assay to identify small molecule agonists of the TGF-beta/Smad signaling pathway	SBE-bla HEK 293T cell lines - B-lactamase under the control of Smad Binding Element. FRET substrate used as reporter fluorescence	13	8598	912	9523	Similar Bioassay as of PID: 588855 but Different Reporter Technique
**5**	1347032	TGF-beta/Smad small molecule antagonists, qHTS assay: Summary	SBE-bla HEK 293T cell lines - B-lactamase under the control of Smad Binding Element. FRET substrate used as reporter fluorescence (Summary)	542	8053	928	9523	Similar Bioassay as of PID: 588855 but Different Reporter Technique
**6**	720537	qHTS for Inhibitors of TGF-b: Hit Validation in HepG2 Cells using COP promoter	HEPG2-cell lines COP-GFP Bioassay for TGF-b inhibitors Copepod's GFP protein used as reporter	55	1358	159	1572	TGF-b inhibitor study. Not Specific to SMAD3
**7**	1347035	TGF-beta/Smad small molecule agonists, qHTS assay: Summary	Summary of multiple studies for agonists of SMAD2 and SMAD3 via multiple assays	8	9404	111	9523	Dataset not specific to SMAD3
**8**	1407233	Inhibition of TGF-beta1/smad3 signaling pathway in human HFL cell extract assessed as decrease in alpha-SMA protein expression at 10 uM after 24 hrs by Western blot analysis	Western Blot Assay (of Lactone Derivatives)	2	-	-	2	Western Blot assay
**9**	1407234	Inhibition of TGF-beta1/smad3 signaling pathway in human HFL cells assessed as decrease in Col1 protein expression level at 10 uM after 24 hrs by Western blot analysis	Western Blot Assay (of Lactone Derivatives)	2	-	-	2	Western Blot assay
**10**	754258	Inhibition of TGF-beta-induced Smad3 phosphorylation in human MDA-MB-231 cells treated 12 hrs before TGF-beta challenge measured after 1 hr by Western blotting	Western Blot Assay (of Curcuminoids)	3	-	-	3	Western Blot assay
**11**	1289250	Inhibition of TGF-beta1-induced Smad2/3 phosphorylation in human LX-2 cells after 1 hr by Western blot analysis	Western Blot Assay (of Furan-carboxylic Acid from fungus Penicillium sp.)	1	-	-	1	Western Blot assay
**12**	1289256	Inhibition of TGF-beta1-induced Smad2/3 phosphorylation in human LX2 cells at 10 uM after 1 hr by Western blot analysis	Western Blot Assay (of Furan-carboxylic Acid from fungus Penicillium sp.)	1	-	-	1	Western Blot assay
**13**	1674917	Inhibition of TGF-beta induced SMAD3 phosphorylation in human A549 cells at 5 to 10 uM pretreated with compound for 2 hrs followed by TGF-beta stimulation by Western blot analysis	Western Blot Assay (of Lactone Derivatives)	2	-	-	2	Western Blot assay
**14**	1058	SMAD Transcription Factor Inhibitors Secondary Dose Response Confirmation (Bioassay)	SMAD3-FoxH1 ELISA based secondary luminescence assay	1	36	-	37	ELISA Assay
**15**	720534	qHTS for Inhibitors of TGF-b: Confirmation of Cherry Picks (Bioassay)	HEPG2-cell lines CAGA-GFP Bioassay	986	119	467	1572	Same Bioassay as of PID: 588855
**16**	720534	HTS of Smad Transcription Factor Inhibitor	Time Resolved – Fluorescence Resonance Energy Transfer (TR-FRET) Bioassay	251	87810	-	88061	TR-FRET Assay
