# Supplementary material for: Probing the binding hypothesis of Smad3 modulators by molecular dynamic simulations for Atherosclerosis Cardiovascular Disease (ASCVD)
Source: PLoS One. 2025 Jun 4;20(6):e0324677. doi: 10.1371/journal.pone.0324677 (PMC12136405; doi:10.1371/journal.pone.0324677)
Supplement: S3 Table — The activity threshold is set to 50% inhibition or above. Compounds have been ranked according to their activity in a descending order. (PDF) [file pone.0324677.s011.pdf]

Name	Structure	Inhibition Potential (%)	Name	Structure	Inhibition Potential (%)
**SM1**	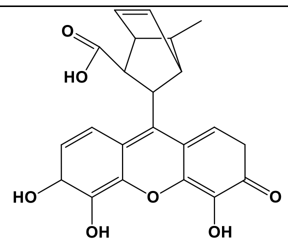	102.17	**SM2**	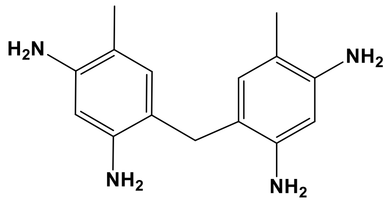	102.14
**SM3**	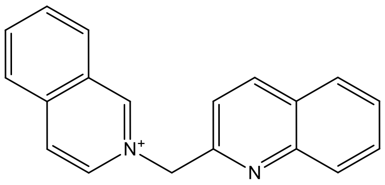	101.08	**SM4**	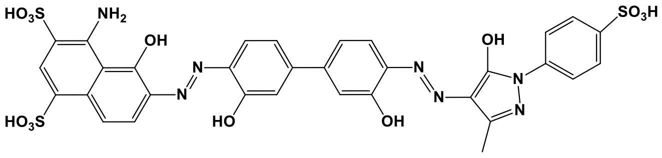	101.01
**SM5**	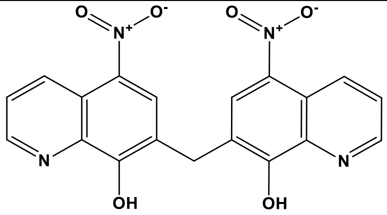	99.29	**SM6**	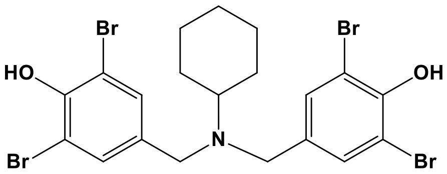	98.67
**SM7**	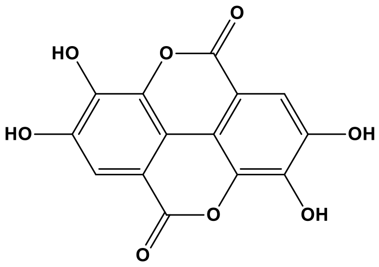	97.61	**SM8**	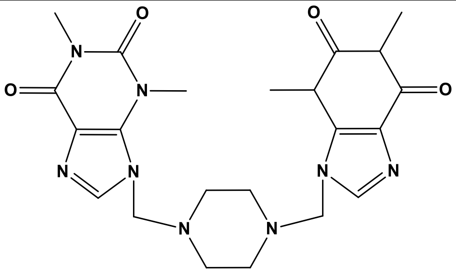	97.58
**SM9**	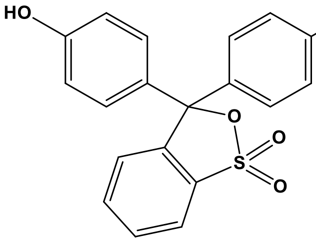	97.01	**SM10**	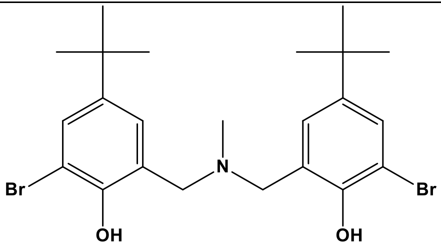	95.66
**SM11**	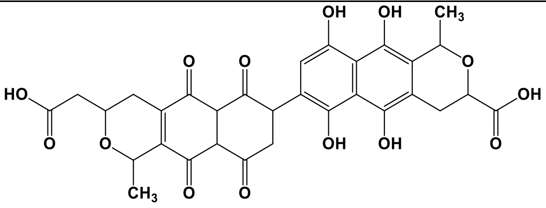	95.55	**SM12**	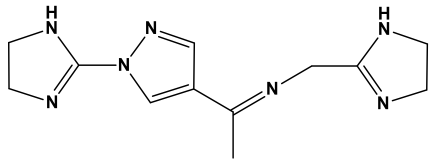	93.16
**SM13**	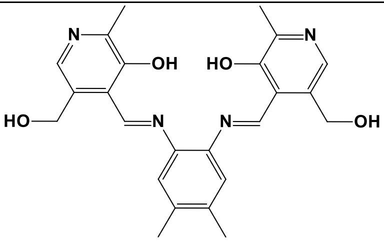	92.98	**SM14**	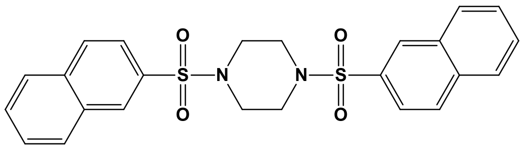	90.55
**SM15**	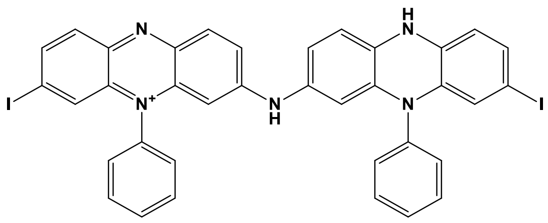	89.73	**SM16**	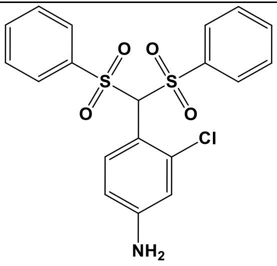	87.14

**SM17**	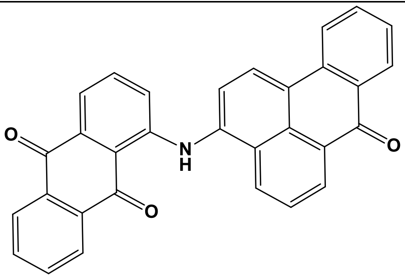	83.11	**SM18**	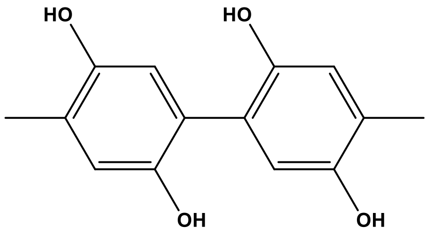	83.07
**SM19**	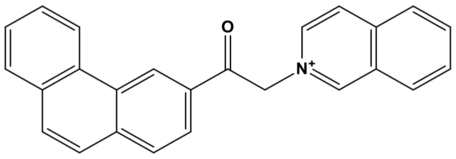	81.26	**SM20**	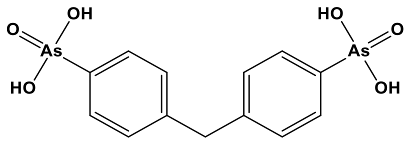	79.24
**SM21**	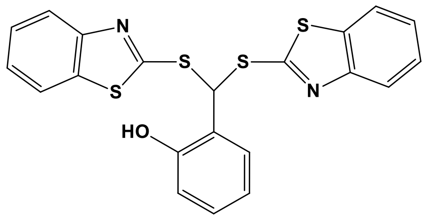	77.95	**SM22**	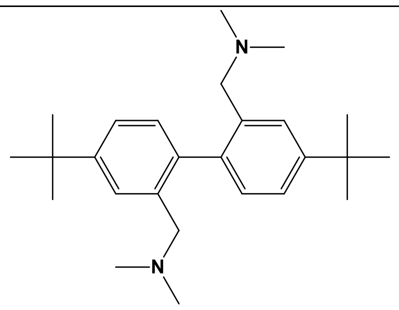	69.89
**SM23**	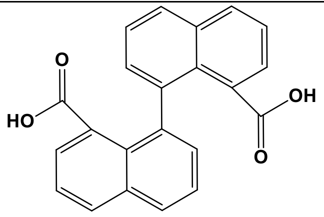	66.63	**SM24**	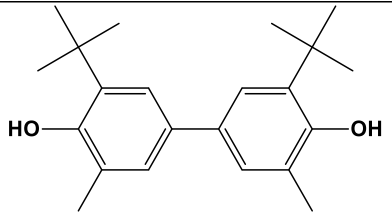	63.43
**SM25**	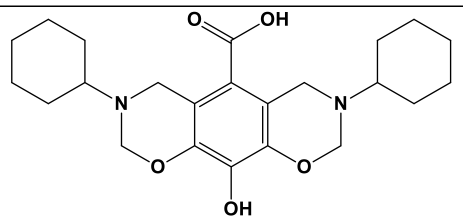	62.4	**SM26**	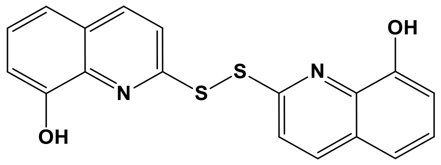	60
**SM27**	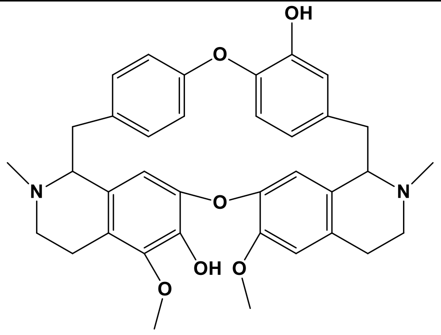	59.4	**SM28**	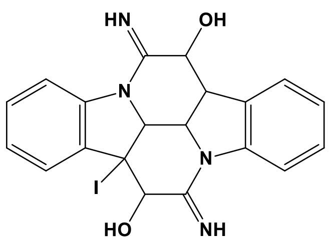	57.11
**SM29**	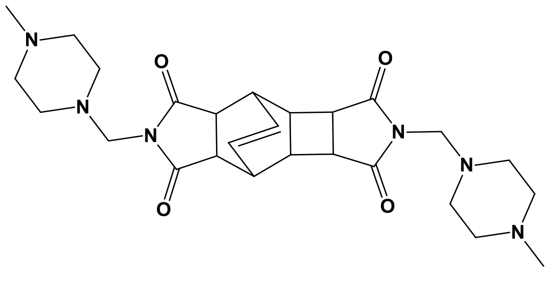	56.55	**SM30**	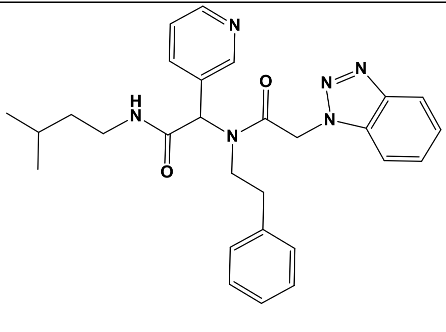	55.42
**SM31**	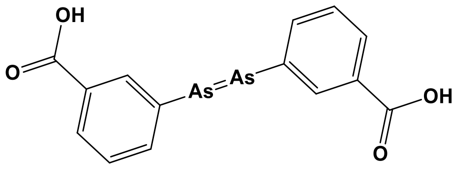	54.69	**SM32**	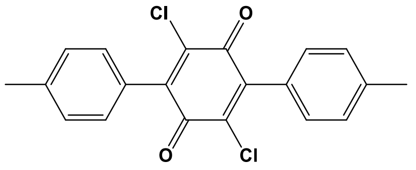	52.18
**SM33**	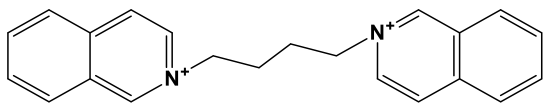	50.59			
