# Supplementary material for: Probing the binding hypothesis of Smad3 modulators by molecular dynamic simulations for Atherosclerosis Cardiovascular Disease (ASCVD)
Source: PLoS One. 2025 Jun 4;20(6):e0324677. doi: 10.1371/journal.pone.0324677 (PMC12136405; doi:10.1371/journal.pone.0324677)
Supplement: S4 Table — Group-I: Ligands with ringed structures around the plane of symmetry at a distance of two-bond length. Group-II: Ligands with ringed structures around the plane of symmetry at a distance of one-bond length. Group-III: Ligands with ringed structures around the plane of symmetry at a distance of three or more bond length. (PDF) [file pone.0324677.s012.pdf]

Group-I	Group-II	Group-III
SM1	SM4	SM6
SM2	SM7	SM8
SM3	SM11	SM10
SM5	SM18	SM12
SM9	SM22	SM13
SM15	SM23	SM14
SM17	SM24	SM16
SM20		SM19
SM25		SM21
SM27		SM26
		SM28
		SM29
		SM30
		SM31
		SM32
		SM33
