# Supplementary material for: Probing the binding hypothesis of Smad3 modulators by molecular dynamic simulations for Atherosclerosis Cardiovascular Disease (ASCVD)
Source: PLoS One. 2025 Jun 4;20(6):e0324677. doi: 10.1371/journal.pone.0324677 (PMC12136405; doi:10.1371/journal.pone.0324677)
Supplement: S5 Table — Revealing the potential of these compounds as drug candidates for ASCVD. The logS of compound should be between close to 0 (0 = water soluble, 0 to −1 = somewhat water soluble, −1 to −4 = slightly water soluble, less than −4 = water insoluble). Intestinal absorption is the percentage of drug absorbed through intestine. Metabolism reveal that out of seven tested CYP isoforms, how many isoforms are inhibited by the tested compound (ideally should be zero). AMES toxicity predicts weather the compound has carcinogenic/mutagenic potential or not. hERG toxicity again reveal the number of hERG channels (out of two tested) being inhibited by the compound. (PDF) [file pone.0324677.s013.pdf]

Name	Water solubility (logS)	Intestinal absorption (percentage)	Metabolism	AMES	hERG
**SM1**	-2.98	82.503	2	No	0
**SM2**	-3.119	75.532	1	Yes	0
**SM3**	-5.375	100	5	Yes	1
**SM4**	-2.892	0.064	0	No	1
**SM5**	-3.809	92.174	5	Yes	1
**SM6**	-3.832	85.408	3	No	1
**SM7**	-2.942	80.608	2	No	0
**SM8**	-2.892	58.318	0	No	0
**SM9**	-3.783	97.001	4	Yes	1
**SM10**	-3.536	88.019	4	No	2
**SM11**	-2.9	38.234	0	No	0
**SM12**	-3.27	80.732	1	No	0
**SM13**	-3.945	70.772	3	No	0
**SM14**	-4.308	98.353	3	Yes	1
**SM15**	-2.923	95.686	3	Yes	1
**SM16**	-4.179	82.17	3	No	1
**SM17**	-3.359	100	2	Yes	1
**SM18**	-2.86	92.178	3	Yes	0
**SM19**	-7.955	98.135	3	No	1
**SM20**	-3.545	45.859	1	No	0
**SM21**	-4.076	89.196	5	No	1
**SM22**	-4.596	89.219	2	No	2
**SM23**	-3.132	98.156	2	No	0
**SM24**	-5.295	91.279	5	No	1
**SM25**	-2.884	91.464	0	No	0
**SM26**	-3.253	90.506	3	No	1
**SM27**	-2.901	92.313	1	Yes	1
**SM28**	-3.074	92.39	3	Yes	1
**SM29**	-2.858	46.807	1	No	0
**SM30**	-4.693	83.742	4	No	1
**SM31**	-3.576	65.943	0	No	0
**SM32**	-5.472	96.787	3	No	0
**SM33**	-4.369	100	2	Yes	1
